# The Evolution of Biomarkers in Thyroid Cancer—From Mass Screening to a Personalized Biosignature

**DOI:** 10.3390/cancers2020885

**Published:** 2010-05-20

**Authors:** Raymon H. Grogan, Elliot J. Mitmaker, Orlo H. Clark

**Affiliations:** Division of Endocrine Surgery, University of California San Francisco, 1600 Divisadero St, C-347, Box 1674, San Francisco, CA 94143, USA; E-Mails: rgroganmd@gmail.com (R.H.G.); ellmit@yahoo.com (E.J.M.)

**Keywords:** thyroid cancer, biomarker, biological marker, molecular marker, tumor marker, molecular pathways, epigenetics, serum based biomarkers, genomics, proteomics

## Abstract

Thyroid cancer is the most common malignancy of the endocrine system. The diagnosis of thyroid nodules, made by neck examination and ultrasonography, is a common event occurring in over 50% of the patient population over the age of 50. Yet, only 5% of these patients will be diagnosed with cancer. Fine needle aspiration biopsy is the gold standard for diagnosing thyroid nodules. However, 10–15% of these biopsies are inconclusive, ultimately requiring a diagnostic thyroid lobectomy. Consequently, research in thyroid biomarkers has become an area of active interest. In the 40 years since calcitonin was first described as the biomarker for medullary thyroid cancer, new biomarkers in thyroid cancer have been discovered. Advances in genomic and proteomic technologies have defined many of these novel thyroid biomarkers. The purpose of this article is to provide a comprehensive literature review of how these biomarkers have evolved from simple screening tests into a complex array of multiple markers to help predict the malignant potential and genetic signature of thyroid neoplasms.

## 1. Introduction

It has been over 40 years since biological markers were first introduced as a way to detect and manage thyroid cancer [1,2]. As the understanding of the molecular pathogenesis of thyroid cancer evolved, the concept of thyroid biomarkers changed. Thus, biomarkers, also known as molecular markers, biological markers, or tumor markers, have become useful not only for detecting thyroid cancer early, but also for detecting recurrent and persistent disease and for predicting the effectiveness of surgical removal, radioiodine ablation, and chemotherapy [3]. 

In theory, biomarkers should tip the scales in favor of early detection. According to the most recent statistics in 2009, the incidence of thyroid cancer accounts for approximately 37,200 cases, yet causes only 0.29% of all cancer deaths [4]. Although death attributed to thyroid cancer is uncommon, because of improvements in imaging techniques, the number of thyroid cancers detected has risen steadily over the past decade [5]. Thyroid cancer is now the sixth most common cancer in women and the second most common cancer in women under 40 years of age [4,6].

Fine needle aspiration (FNA) cytology remains the procedure of choice for preoperatively establishing the diagnosis of a thyroid nodule. Despite the benefits of FNA cytology for diagnosing papillary, medullary, and anaplastic thyroid cancer, it is not helpful in determining whether follicular or Hurthle cell thyroid tumors are benign or malignant. Also some FNA cytology results suggest, but do not definitively diagnose, papillary thyroid cancer. Consequently, the management of the indeterminate thyroid follicular nodule and the suggestive but non-diagnostic papillary nodule continues to be suboptimal. The idea of identifying a single genetic mutation or cell surface marker that would distinguish a benign thyroid follicular nodule from that of a thyroid carcinoma or clarify the diagnosis of papillary thyroid carcinoma is still elusive. This is because biomarkers can encompass a spectrum of a thyroid follicle cell’s physiological state, *en route* to its transformation from a benign to a malignant state [3]. The recent multistep model of thyroid follicular carcinogenesis, based upon Vogelstein’s model for colon cancer [7], is an excellent model to study the role of biomarkers to improve thyroid cancer detection. This model also provides a firm basis for the selection of patients who can be safely observed or who need lobectomy or total thyroidectomy. 

Molecular techniques as diagnostic tools are rapidly changing. Two of the earliest biomarkers discovered in thyroid cancer stemmed from the seminal work that established serum assays for measuring serum thyroglobulin and calcitonin levels [8,9]. Their discovery helped revolutionize the management of patients with thyroid cancer in terms of earlier diagnosis and more sensitive follow-up. Biomarker studies have expanded to include genetic mutations and molecular changes to detect some of the earliest events in thyroid tumorigenesis. As technology improved, high-throughput genomic and proteomic assays have become the latest methods for identifying a multitude of biomarkers to reflect a molecular profile signature for each tumor type at any given stage [3]. This article reviews the literature on thyroid cancer biomarker research as it has evolved over time, beginning with serum-based biomarkers, followed by mutation-based biomarkers, and ending with the more current genomic and proteomic-based biomarker profiles. Each of the following sections is a synopsis of the most clinically significant thyroid cancer biomarkers to date. These sections are not meant to be exhaustive or all encompassing lists. Instead, they are a starting point for understanding each type of biomarker and its utility in the field of thyroid cancer research.

## 2. Serum-Based Biomarkers

Serum biomarkers represent the first generation of thyroid biomarkers. Ideally, a serum biomarker is one that is highly sensitive and specific, can establish diagnostic certainty, and can be easily measured [10]. This definition has remained fairly consistent over several decades, despite the introduction of complex bioinformatics systems that are being used as analytical tools to identify new biomarkers. Herberman [11] proposed five criteria for a biomarker to be useful in cancer detection: (1) measurement should be simple, reproducible, easily available and cost effective; (2) should detect a quantitative difference so that it can distinguish those with or without disease; (3) should have high sensitivity; (4) should be able to monitor for recurrence of disease, and (5) should have high specificity. 

### 2.1. Calcitonin

Calcitonin, an anti-hypercalcemic hormone secreted by the parafollicular C-cells of the thyroid, fulfills the above-mentioned criteria as a serum-based marker for medullary thyroid cancer (MTC) [12]. In conjunction with FNA, calcitonin is a biomarker that helps optimize the sensitivity and specificity of diagnosing MTC. However, it is not a foolproof test, and calcitonin measurements have differed depending on the assays used [13]. Radioimmunoassays were initially used to detect calcitonin in the circulation. Because these assays used polyclonal antibodies, several different monomers of calcitonin could be detected, making the test less specific [14]. However, studies have demonstrated that both immunoradiometric assays and immunochemiluminescent assays, which are based on monoclonal antibodies, can eliminate variation and improve reliability and sensitivity in the measurement of calcitonin [15,16]. In addition, increased calcitonin levels are not exclusive to MTC, as they have been reported in other conditions such as C-cell hyperplasia, thyroid nodules of follicular cell origin, increasing age, increased body-mass index, cigarette smoking, breast feeding and small cell carcinoma of the lung [15,17]. However, hypercalcitonemia in the above-mentioned conditions occur with a prevalence of less than 5% [15]. 

The role of calcitonin as a screening marker for MTC has shifted from a “mass-screening” paradigm to use for specific situations. For instance, there are two types of medullary thyroid cancer: a familial (~25%) type and a sporadic (~75%) type. The level of calcitonin in MTC is associated with an increasing tumor burden as evidenced by a large tumor size along with the presence of lymph node or distant metastasis [18,19]. Some rare patients with poorly differentiated or metastatic MTC have relatively low calcitonin levels due to cellular heterogeneity and their tumors stain less well for calcitonin and more for carcinoembryonic antigen (CEA). These patients have more aggressive disease [20]. Overall, calcitonin is more sensitive for documenting recurrent tumor but CEA levels are better predictors of tumor aggressiveness. Provocative stimulation tests using calcium or pentagastrin increase the sensitivity of calcitonin. Provocative stimulation of calcitonin, has in general been replaced by genetic testing using *RET* mutations because the latter is more sensitive and specific [21,22].

### 2.2. Thyroglobulin

Serum thyroglobulin is a glycoprotein that is synthesized by thyroid follicular cells and is the precursor molecule for the production of thyroxine (T4) and triiodothyronine (T3) [23]. Thyroglobulin is made by normal, hyperplastic and neoplastic thyroid tissue. Thyroglobulin is a valuable serum marker for detecting recurrent or persistent well-differentiated thyroid cancer of follicular cell origin, as there should be no thyroglobulin present after a total thyroidectomy unless residual thyroid tissue is present [24,25]. Although mainly localized in the colloid, thyroglobulin can be resorbed into the peripheral circulation, allowing it to be detected in the patient’s serum. Van Herle initially developed an assay that measured serum thyroglobulin using a double radioimmunoassay technique [8]. As is true for the serum detection of calcitonin, advancements are continually being made that improve both the sensitivity and specificity of serum thyroglobulin detection [24]. However, problems still exist with the interpretation of these assays, given the presence of certain conditions. For example, the presence of anti-thyroglobulin antibodies interfere with blood thyroglobulin levels often with inappropriately low and rarely falsely high thyroglobulin levels [26,27]. Thyroglobulin assays are not as reliable when patients are on thyroid hormone suppressive therapy. In addition, there are many benign thyroid conditions (e.g., thyroiditis, thyrotoxicosis, benign adenoma and iodine deficiency) that may cause a false-positive reading, as reflected by increased thyroglobulin levels [28]. For thyroglobulin to serve as a reliable and sensitive marker for recurrent or persistent differentiated thyroid cancer, two conditions must be met. First, the patient must undergo total (or near total) thyroidectomy and then receive radioiodine ablation of the remaining thyroid remnant so as to assure that any subsequent thyroglobulin measurements are due to recurrent thyroid cancer and not persistent normal thyroid tissue. Second, to improve the sensitivity of measured serum thyroglobulin, thyroid stimulating hormone (TSH) stimulation must occur either through thyroid hormone withdrawal or by administering recombinant human TSH (rhTSH) [23]. Unfortunately, even if all the above-mentioned criteria are met, thyroglobulin levels although generally very helpful in documenting tumor recurrence, may still rarely be undetectable in the serum when there is known residual disease [24]. This is most likely to occur in patients with elevated antithyroglobulin antibodies.

The serum-based biomarkers, calcitonin and thyroglobulin, provided the impetus for future research in the discovery of biomarkers in thyroid cancer. Though relatively useful as initial tests, calcitonin and thyroglobulin have several limitations. More recently, molecular studies using reverse transcriptase polymerase chain reaction (RT-PCR) have been used to measure tissue-tumor specific messenger RNA levels in the circulation. Both thyroglobulin (Tg) mRNA and TSH receptor mRNA have been extensively studied. Initially, the sensitivity and specificity of both were questioned because they were routinely found in the blood of healthy subjects. Some of the explanations for this included ectopic transcription of thyroglobulin mRNA in lymphocytes and alternative splicing of thyroglobulin mRNA in thyroid follicular cells [29,30]. Other researchers have found that primer selection was an integral step in determining that Tg mRNA and TSHr mRNA are highly sensitive and specific markers for detecting thyroid cancer recurrence in patients who are on thyroid hormone suppressive therapy or who have circulating antithyroglobulin antibodies [25,31]. 

The first generation of biomarkers, the serum-based biomarkers, has helped improve the management of patients with thyroid cancer, but further advances are necessary. As the understanding of thyroid carcinogenesis continues to unfold, it is becoming clear that the single serum biomarker approach is too simplistic.

## 3. Mutation Based Biomarkers

The next step in the evolution of thyroid cancer biomarkers was the study of genetic mutations in thyroid tumors. Genetic alterations in thyroid tumors can be divided into two categories, inheritable (germline) mutations, and sporadic (somatic) mutations. Investigations into the inheritable and sporadic mutations in thyroid cancer have proceeded in parallel with one another. Although many gene mutations have been studied, only one inheritable genetic mutation and five to eight sporadic mutations are currently of significance (Table 1). The single known inheritable gene mutation associated with thyroid cancer is a point mutation in the *RET* proto-oncogene that causes medullary thyroid cancer. The association of a genetic mutation with medullary thyroid cancer was first hypothesized in the late 1980s, but was not specifically identified until 1993 [32]. The first sporadic mutation identified in thyroid cancer was described in 1987 and involved a genetic defect in the RAS protein family [33]. In 1990, somatic *RET/PTC* translocations were identified in papillary thyroid cancer. In 1992, *P53* mutations in anaplastic thyroid cancer and *NTRK1* mutations in papillary thyroid cancer were identified [34,35,36]. There was a subsequent lull in the discovery of genetic mutations in thyroid cancer until the year 2000, when *PAX8/PPARgamma* translocations were found in follicular thyroid cancers [37]. This was followed by the discovery of *BRAF* mutations, first in melanoma, then in papillary thyroid cancer in 2003. *BRAF* mutations are the most common somatic mutations in papillary thyroid cancer and are possibly one of the most significant genetic mutation findings in thyroid cancer research [38]. Several investigators have continued to search for the genetic mutation responsible for familial non-medullary thyroid cancer, but the responsible genetic mutation has not yet been identified.

**Table 1:** Frequency of Sporadic Mutations in Thyroid Cancer by Phenotype.

Mutation	Adenoma	PTC	FTC	Anaplastic	Ref.
BRAF	0%	44%	<1%	24%	[39]
RET/PTC	Controversial	35%	0%	0%	[40]
RAS	13%	10%	40%	22%	[41]
PAX8/PPARδ	11%	0%	36%	0%	[42]
P53	0%	1%	1%	55%	[43]
NTRK1	Unknown	12%	Unknown	Unknown	[44]
PTC: papillary thyroid cancer, FTC: follicular thyroid cancer					

As the understanding of thyroid tumor formation advanced, the focus of mutation-based biomarkers shifted from single genetic mutations to molecular signatures and panels of multiple mutations. This shift in approach happened because investigators realized that the progression from a normal cell to a tumor is a complex process that likely involves multiple genetic and possibly epigenetic events, making identification of a single mutation responsible for sporadic thyroid cancer unlikely. Instead, a spectrum of many different mutations might be responsible for sporadic thyroid cancer. The discovery of single genetic mutations remains important for understanding cancer formation, but single mutations are not practical clinical biomarkers for thyroid cancer. Panels of DNA mutations are being explored as thyroid cancer biomarkers. Another area of interest is in microsatellite instability, which can be used as a biomarker for cancer development. 

### 3.1. BRAF

**Figure 1:**
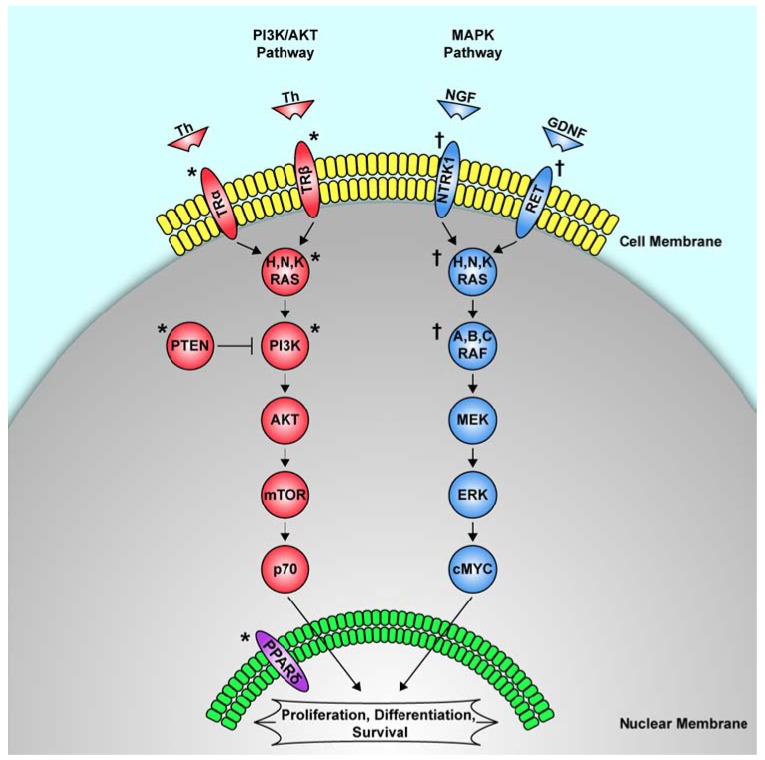
Genetic Mutation Pathways Associated with Sporadic Thyroid Cancer. Sporadic thyroid cancer is thought to occur via three independent cellular pathways based on the type of sporadic genetic mutation that leads to the development of thyroid cancer.

† Denotes known genetic mutations associated with sporadic *papillary* thyroid cancer. Mutations of the MAPK pathway (in blue) are associated with papillary thyroid cancer. * Denotes known genetic mutations associated with sporadic *follicular* thyroid cancer. Sporadic mutations of the PI3K/AKT pathway have been associated with follicular thyroid cancer. In the third pathway, mutations of the nuclear receptor PPARgamma have been associated with follicular thyroid cancer. Th = thyroid hormone, TR = thyroid hormone receptor, NGF = nerve growth factor, GDNF = glial cell line-derived neurotrophic factor.

BRAF is part of the RAF family (A, B, and C) of serine/threonine kinases. These proteins relay signals from membrane-bound receptors to downstream regulators of the MAPK pathway that ultimately control the expression of several genes responsible for cell proliferation, differentiation, and apoptosis (Figure 1). Most *BRAF* mutations are caused by a single point mutation that replaces valine for glutamic acid at position 600, and is designated *BRAF*^V600E^. The *BRAF*^V600E^ mutation is currently the most common genetic mutation found in papillary thyroid cancer (29–83%) [39]. It is also frequently found in the tall-cell variant of papillary thyroid cancer, poorly differentiated thyroid cancer, and undifferentiated thyroid cancer of papillary origin. It has rarely been found in follicular thyroid neoplasms [45]. Another activating *BRAF* mutation, *BRAF*^K601E^, has been found in thyroid adenomas and the follicular variant of papillary thyroid cancer [46,47]. Some, but not all, investigators have found that activating *BRAF* point mutations confer a high rate of transformation to malignancy, and have been associated with later age at presentation, extra-tumoral extension, lymph node metastasis, and a higher rate of recurrence [45,48]. A recent meta-analysis of 1168 patients confirmed an association between the *BRAF*^V600E^ mutation and higher clinical staging as well as increased extratumoral invasion [49,50]. As a biomarker, *BRAF* is almost exclusively found in papillary thyroid carcinomas, and can be used as a marker for this tumor type. *BRAF* is also thought to confer a poorer prognosis compared to sporadic thyroid tumors that do not have the mutation, but for this to be significant in clinical decision-making, more prospective studies need to be done.

### 3.2. RET/PTC Rearrangements (Sporadic Papillary Thyroid Cancer)

*RET* mutations that cause sporadic papillary thyroid cancer are rearrangements rather than point mutations like the ones found in familial medullary thyroid cancer. *RET* gene gain-of-function happens when the *RET* gene is rearranged and connected to a new promoter from a gene that is constitutively expressed in thyroid follicular cells. This leads to uncontrolled downstream signaling and activation of the MAPK pathway. At least 15 separate genes are known to rearrange with the *RET* gene, causing mutations that are responsible for papillary thyroid cancer. In fact, these mutations are almost exclusively found in papillary thyroid cancer. The *RET/PTC* 1 and 3 rearrangements are by far the most common. A high frequency of *RET/PTC* rearrangements have been found in papillary thyroid cancers after radiation exposure, both from nuclear accidents, such as Chernobyl, and from exposure to external beam radiation therapy. *RET/PTC* rearrangements are also common in childhood papillary thyroid cancer. A high frequency of *RET/PTC* rearrangements have also been identified in papillary microcarcinomas, suggesting that they are an early event in the formation of papillary thyroid cancer. The *RET/PTC* rearrangements are important for understanding how sporadic thyroid cancer occurs, but are not useful as clinical biomarkers.

### 3.3. RET Point Mutations (Familial Medullary Thyroid Cancer)

The *RET* gene, first identified as a proto-oncogene in 1985 [51], encodes the RET receptor: a plasma membrane bound tyrosine-kinase, which is expressed in neuroendocrine and neural cells. This means that the *RET* gene product is expressed in normal thyroid parafollicular or c-cells, but not in normal thyroid follicular cells. Multiple endocrine neoplasia type 2A (MEN 2A) was assigned to chromosome 10 by linkage analysis in 1987, when the location of the *RET* gene was still unknown [52]. The following year, the nucleotide sequence of the *RET* gene was determined and in 1989, the *RET* gene was mapped to chromosome 10q11.2 [53,54]. In 1991, a linkage analysis of MEN 2A patients narrowed the linkage to chromosome 10q11.2, which at that time was known to be the location of the *RET* gene as well [55]. This finding was confirmed in 1993, the same year that specific germline mutations of the *RET* gene were found in families with MEN 2A, which definitively established the association of *RET* point mutations on chromosome 10q11.2 with the development of medullary thyroid cancer [32,56,57]. These mutations were also linked to MEN 2B patients and to families with familial medullary thyroid cancer who develop medullary thyroid cancer without other endocrinopathies. 

Point mutations of the *RET* gene that cause medullary thyroid cancer result in a gain of function of the RET receptor. Loss-of-function mutations of the *RET* gene do not cause thyroid cancer, but instead cause Hirchsprung’s disease and congenital megacolon. Multiple individual *RET* gene point mutations have been identified and have led to the genotype-phenotype correlation of *RET* mutations and the stratification of hereditary medullary thyroid cancer into three risk groups (Levels 1, 2, and 3). These levels correlate with the genotype as well as with the age of onset and the level of aggressiveness of thyroid cancer, with Level 1 mutations being the least aggressive and Level 3 mutations being the most aggressive. Because the penetrance of thyroid cancer is nearly 100% in patients with *RET* point mutations, prophylactic thyroidectomy is now a standard of care in all patients with these mutations. Current recommendations call for prophylactic thyroidectomy between the ages of five and 10 for individuals with Level 1 mutations, at no later than five years of age for Level 2 mutations, and at diagnosis or no later than six months of age for Level 3 mutations [58,59]. Earlier operation is indicated for these patients if they are not serum calcitonin-negative and thyroid node negative by ultrasound. Genetic testing, because it is more sensitive and specific, has replaced calcitonin monitoring to detect which patients are susceptible to developing familial medullary thyroid cancer [60]. If the serum calcitonin level is increased or there are thyroid nodules by ultrasound then surgery is indicated. The hereditary *RET* point mutations are the most specific biomarkers in clinical use today for diagnosing patients who will develop medullary thyroid cancer. No other currently used thyroid cancer biomarker is as sensitive or specific.

### 3.4. RAS

The products of the *RAS* gene family (H, N, and K) are a group of intracellular proteins that relay signals from activated membrane-bound receptors to downstream kinases of the MAPK pathways that regulate cell proliferation, differentiation, and cell survival (Figure 1). Up to 30% of human tumors are thought to have *RAS* mutations. Activating point mutations in the *RAS* genes produce constitutively active RAS proteins that cause uncontrolled activation of their downstream cellular control pathways, leading to aberrant cell proliferation and differentiation. 

In thyroid tumors, the most frequent *RAS* mutations are found in the *N-RAS* gene, followed by *H-RAS*, and least frequently, *K-RAS*. *RAS* mutations are found in a wide variety of thyroid tumors including follicular adenomas, follicular carcinomas, papillary carcinomas, poorly differentiated carcinomas, and undifferentiated carcinomas. They are more frequently associated with follicular tumors than papillary tumors. Because *RAS* mutations are found in the entire spectrum of thyroid cancers, and with increasing frequency as tumors become more undifferentiated, *RAS* mutations have been suggested to be a biomarker for a more aggressive form of thyroid cancer [61]. However, *RAS* mutations cannot be used to reliably differentiate between benign and malignant tumors. *RAS* mutations do seem to confer an increased risk of uncontrolled proliferation, which predisposes the cells to accumulation of other genetic defects and eventual tumor formation. As a biomarker, this means that a benign follicular tumor with a *RAS* mutation may have a higher likelihood of subsequently transforming into a follicular carcinoma, but the exact risk is not known.

### 3.5. PAX8/PPARgamma

PPARgamma is a nuclear receptor involved in cell cycle control and apoptosis. PAX8 is a transcription factor involved in the development and regulation of thyroid-specific genes. An abnormal translocation causes a fusion of the *PAX8* promoter with the *PPARgamma* gene, forming the mutated PAX8/PPARgamma fusion protein (PPFP), which causes inactivation of the wild-type PPARgama gene. *PAX8/PPARgamma* translocations are found predominantly in follicular thyroid carcinomas. However, they have also been found, with a much lower frequency, in follicular adenomas and papillary carcinomas. *PAX8/PPARgamma* mutations are typically associated with follicular thyroid cancers that present at an earlier age and with a high frequency of vascular invasion. This predisposition to early vascular invasion means that few tumors with these mutations are found in the pre-malignant state, which is why few follicular adenomas are found with the mutation. As a biomarker, a *PAX8/PPARgamma* rearrangement is a strong indicator that a tumor is a follicular carcinoma with an early propensity for vascular invasion, but it is not sensitive or specific enough to be used on its own to reliably distinguish between benign and malignant follicular neoplasms. However, if a *PAX8/PPARgamma* mutation is found in a follicular neoplasm, there is a higher likelihood that the lesion is or will progress to a follicular cancer [62]. 

### 3.6. P53 Tumor Suppressor

P53 is an important tumor suppressor that regulates cell cycle arrest and apoptosis. When a cell is damaged, p53 is responsible, via a cascade of downstream regulators, for either halting the cell cycle to allow DNA repair, or for initiating apoptosis in severely damaged cells. In these ways, p53 acts to prevent damaged cells from converting into tumors. Mutations that cause inactivation of the *P53* gene may be found in up to 50% of all human malignancies, making it the most common mutation associated with human cancer [63]. *P53* mutations are common in poorly and undifferentiated thyroid cancers and in thyroid cancer cell lines, but are rare in well-differentiated thyroid cancers. This pattern of distribution suggests that an initial inciting event destabilizes the thyroid cell DNA, causing thyroid cancer formation, and after that initial event, *P53* mutations cause the thyroid cancer to progress to an undifferentiated state [64]. This theory helps to explain the high prevalence of *P53* mutations in anaplastic thyroid cancer compared to well-differentiated thyroid cancer. There is also evidence that p53 inactivation caused by events other than mutations may play a role in well-differentiated thyroid tumors [65,66]. Because of their high incidence in undifferentiated thyroid cancer, the presence of *P53* mutations may be predictive of a highly aggressive thyroid cancer, but on its own, sensitivity is too low to be used as a reliable clinical biomarker.

### 3.7. NTRK1

The neurotrophic tyrosine kinase type 1 (*NTRK1*) gene encodes a tyrosine kinase cell surface receptor known as NTRK1 that binds its ligand nerve growth factor (NGF). After binding to NGF, the NTRK1 receptor activates the RAS proteins, which in turn are responsible for signaling via the MAPK pathway and controlling cell proliferation, differentiation, and apoptosis (Figure 1) [44]. In the mid 1990s, three genes, tropomyosin 3 (TPM3), translocated promoter region (TPR), and TRK fused gene (TFG), were found to rearrange with *NTRK1* to form translocations that are associated with papillary thyroid cancer [36,44]. The incidence of *NTRK1* translocations is exclusive to papillary thyroid cancer, but with a much lower frequency (5–15%) than *RET* translocations or *BRAF* point mutations. The few studies that have been done on the prognosis of *NTRK1* translocations seem to show that they have a worse prognosis than *RET* translocations [67]. However, data on NRTK1 incidence and prognosis is limited, making *NTRK1* a poor clinical biomarker at this time.

### 3.8. DNA Mutation Panels

Papillary and follicular thyroid cancers are hypothesized to develop via distinct mutation pathways, with more than 70% of papillary thyroid cancers caused by mutations of the RET/RAS/BRAF/MAPK pathway, and more than 80% of follicular thyroid cancers caused by mutations of the *RAS* genes or *PAX8/PPARgamma* translocations [68]. In most thyroid cancers, these mutations are mutually exclusive events, meaning only one of these mutations is found in any particular cancer [38]. When these mutations are used as independent biomarkers their sensitivity and specificity are too low to be clinically relevant. But, because almost 70–80% of thyroid cancers should have at least one of these mutations, a panel of all the mutations may be able to improve the diagnostic accuracy of thyroid tumor FNA cytology. In 2009, a prospective study was completed of 470 thyroid nodule FNAs using a diagnostic panel of DNA mutation testing of all these genes [68]. This mutation-based diagnostic panel found that if any one of these mutations (*RET*, *RAS*, *BRAF*, *MAPK*, *PAX8/PPARgamma*) were detected in an FNA sample, there was a 97% chance that the nodule was malignant. The panel also demonstrated 100% sensitivity and 100% specificity in diagnosing FNA samples of “indeterminate” cytology as either benign or malignant. However, the sensitivity of the panel decreased to 75% for FNA samples classified as “follicular neoplasm”, and 60% for FNA samples classified as “suspicious for malignancy”. Up to 30% of thyroid cancers will have no currently known mutations, thus further decreasing the utility of current DNA mutation panels. Signatures from gene expression profiles will eventually be used to construct new DNA mutation panels for FNA-based diagnosis of thyroid nodules. 

### 3.9. Microsatellite Instability

Microsatellites are simple DNA sequences of two to five nucleotide base pairs that are repeated multiple times. These repeated sequences are found scattered throughout the genome, and are a normal part of the genetic makeup. Microsatellites are useful as highly specific DNA markers in genetic analysis because they are highly polymorphic and normal variation occurs between individuals. In 1993, it was discovered that variations in the length of microsatellites (microsatellite instability) at individual loci could be used as a marker of DNA mutation and instability in colorectal cancer [69]. Microsatellite instability was eventually determined to occur secondary to mutations of DNA mismatch repair genes. The defective mismatch repair genes cause the microsatellites to be replicated incorrectly. More importantly, these defective repair genes allow accumulation of multiple somatic point mutations that eventually lead to tumor formation. In this way, microsatellite instability can be used as a genetic signature that identifies cells at risk for becoming malignant, and can possibly allow for early intervention. Since the first study demonstrating microsatellite instability in thyroid lesions in 1997 [70], small panels of microsatellites have been studied in thyroid cancer, with varying outcomes. Microsatellite instability has been demonstrated in both papillary and follicular thyroid tumors, as well as in benign thyroid adenomas [71,72]. However, others have been unable to find any microsatellite instability in thyroid tumors [73]. One problem with studying microsatellite instability is deciding which microsatellites to include in the analysis. High-throughput studies of the entire genome that would generate a genetic signature have not been done, but are needed to better determine the clinical utility of microsatellite instability in thyroid cancer. Currently, microsatellite instability cannot be used as a clinical biomarker for thyroid cancer because its frequency in various types of thyroid tumors is still debatable.

## 4. Epigenetic Biomarkers

Epigenetics is defined as the study of heritable changes in gene expression that occur independent of changes in the primary DNA sequence. Mechanisms of epigenetic regulation include DNA methylation, histone protein modification, nucleosome positioning, and microRNA silencing. In thyroid cancer, DNA methylation, histone modifications, and microRNA silencing have all been studied, but there is minimal data on nucleosome positioning. All these mechanisms regulate DNA by silencing the expression of genes when they are activated. Defects in these mechanisms lead to aberrant gene expression and both have been implicated in multiple human cancers. One of the most exciting aspects of epigenetic regulation is that unlike genetic mutations, these processes are easily reversible with various therapeutic agents. Because of this, markers of epigenetic causes of thyroid cancer have been investigated. As with the other thyroid cancer biomarkers, work has proceeded from initially identifying these changes in thyroid cancer, to identifying epigenetic “signatures” that can predict cancer formation before the cancer becomes clinically apparent so that treatment can begin at an early stage.

### 4.1. Hypermethylation

DNA methylation is an epigenetic regulatory mechanism involved in silencing gene expression that is particularly important in normal embryogenesis. It occurs by adding a methyl group to the cytosine residue of a CpG dinucleotide. Regions of DNA that contain multiple copies of CpG dinucleotides are termed CpG islands and are usually located at the 5’ end of gene promoters. Gene silencing after methylation of a CpG island occurs by either blocking the binding of transcription factors to the promoter region or by recruitment of methyl-binding DNA transcription repressors to the promoter. Aberrant methylation, or hypermethylation, of tumor suppressor genes has been identified in many human tumors including thyroid tumors [74]. Hypermethylation of multiple genes has been identified in association with the PIK3/AKT pathway in follicular thyroid cancers, and of the MAPK pathway in papillary thyroid cancers [75,76]. Hypermethylation has also been identified in benign thyroid tumors, though to a lesser extent than in thyroid carcinomas. Investigations have also linked *BRAF* mutations to hypermethylation, which may be associated with increased production of vascular endothelial growth factor in *BRAF*-positive thyroid tumors [77]. Other genes identified as hypermethylated in thyroid cancer include the *TSH* receptor, *NIS*, *PCNA*, *CDKN2A*, *FGFR2*, and *RASSF1A*. High-throughput technology has shown that patterns of methylation that correlate with tumor formation and progression produce a genetic signature called the CpG island methylator phenotype (CIMP). The CIMP signature confers a high degree of genetic instability and has been identified as an early marker of progression from benign to malignant neoplasia in colon cancer [78]. A CIMP signature has not been identified in thyroid cancers, but is the next step in the evolution of hypermethylation as a thyroid cancer biomarker.

### 4.2. MicroRNA

MicroRNAs, also known as miRNAs or miRs, were discovered in 1993, and since then, more than 700 human miRNAs have been catalogued [79,80]. They are single-stranded RNAs of 20–25 nucleotides that down-regulate gene expression at the post-transcriptional level via translational repression, mRNA cleavage, and mRNA decay. They can act as either oncogenes or tumor suppressors and have been identified in the regulation of a wide variety of functions including hematopoiesis, cell proliferation, cell survival and apoptosis [81]. A single miRNA can regulate the expression of hundreds of different genes. The first study of miRNA expression in thyroid cancer was in 2005, and found 17 miRNAs that were over expressed and six miRNAs that were under expressed [82]. Most studies on thyroid cancer miRNA expression are in papillary thyroid cancer and have consistently shown upregulation of miRNAs 146b, 221, 222, 224, 155, and 181b. Fewer studies have been done on follicular thyroid cancers, but upregulation has been identified in miRNAs 155, 187, 221, 222, and 224 [83]. As is true for genomics, miRNA “signatures” are beginning to be identified, which may prove useful for diagnosing thyroid cancer from FNA samples. A few small prospective trials using FNA samples have established miRNA as a possible diagnostic tool, but larger clinical trials are needed to confirm the utility of miRNAs as a clinical biomarker [84]. 

## 5. Genomics

The term genomics was first used in 1987 and refers to the study of the structure and organization of a genome of a particular organism. This includes the mapping and sequencing of the genome, as well as the analysis of the information gained from mapping and sequencing in the context of their biological significance and biomedical application [85]. Technology is expanding the study of genomics and our understanding of the genome at an almost exponential pace. A full review of genomics, even in the context of thyroid cancer, is beyond the scope of this review. The following general overview is intended as a starting point for understanding the important findings in the field of genomics as they relate to thyroid cancer. 

Initially, the field of genomics was focused on mapping the human genome and understanding the effects of single genes in a system. The sequencing of the human genome, along with the development of high throughput technologies, have shifted the focus of the field of genomics to gene expression profiling and the quest to identify genetic signatures of disease. Many different gene expression profiling technologies are currently in use, including cDNA microarrays, oligonucleotide arrays, and Serial Analysis of Gene Expression (SAGE) [86,87,88]. These technologies allow the simultaneous study and comparison of the expression of thousands of genes in varying conditions. The utility of this approach is in recognizing patterns of gene expression to gain insight into biological processes rather than focusing on individual gene expression. These technologies are being used in cancer research to elucidate the mechanisms of tumor formation to identify a patient’s risk of developing cancer, to predict prognosis once cancer has developed, and ultimately to design targeted therapies for cancer treatment. The hope is that gene expression profiling will someday lead to personalized medicine, in which a DNA signature unique to each patient can be developed and allow tailoring of treatment specific to that patient’s needs.

One of the first gene expression profiles done in thyroid cancer was on a group of 8 papillary thyroid cancers in 2001 [89]. This study identified a papillary thyroid cancer gene signature that consisted of more than 220 differentially expressed genes, many of which were previously identified as being differentially expressed in papillary thyroid cancer, and many of which were not. In 2005, a DNA microarray analysis on papillary thyroid cancer identified genetic signatures that distinctly correlate with mutations in *BRAF*, *RAS*, and *RET/PTC* papillary thyroid cancers [90]. These genetic signatures support the theory that sporadic papillary thyroid cancers originate via mutations in the RET/RAS/BRAF/MAPK pathway, and could also predict tumors with these mutations with high accuracy. This study also identified specific genetic signatures that distinguish classic papillary thyroid cancer from the tall cell and follicular variants. 

Other gene expression profile studies have identified distinct genetic signatures that correlate with the *PAX8/PPARgamma* translocation in follicular thyroid cancer, and in *RET/PTC* rearrangements in radiation-induced papillary thyroid cancer, and can help predict an increased risk of invasion in papillary thyroid cancer [91,92,93]. Multiple gene expression profiling studies on thyroid cancer have been completed in a relatively short period of time. This is reflected in a 2006 meta-analysis of 21 gene expression profile studies [94]. The meta-analysis highlights both the strengths and weaknesses of high throughput gene expression profiling. The analysis identified a small group of genes that were consistently differentially expressed in multiple studies. Within this group were a subset of genes known to be involved with thyroid cancer, and a subset of genes that had previously not been identified in relation to thyroid cancer. However, several genes identified as being involved in thyroid cancer in individual studies were not consistently found across multiple studies. This highlights the fact that gene expression profiling is still an evolving field with many different and complicated technologies. Because these studies generate such large amounts of data, complex mathematical formulas and statistical analyses are required. Currently, the analysis of high-throughput gene expression profile studies has not been standardized, which might be partially responsible for the occasional contradictory findings associated with these studies. Even though these studies have already produced many exciting findings regarding the development and biology of thyroid cancer formation, there is currently not enough concordance of these genetic signatures to use them as biomarkers for diagnosis or prognosis of patients with these tumors.

## 6. Proteomics

Proteomics is defined as the study of protein structure and function. The term was first introduced as an analogy to “genomics,” but in this case referring to the entire protein spectrum [95]. Before high-throughput proteomics was developed, protein expression was studied by immunohistochemistry, serving as an adjunct to FNA cytopathology, hoping to improve the diagnostic accuracy in indeterminate thyroid nodule FNA samples. Immunohistochemical markers have been used to determine the molecular phenotype of these indeterminate lesions. 

### 6.1. Immunohistochemistry

Several immunohistochemical markers representing different components of the cell, such as the membrane, the cytoplasm, or the nucleus, have been studied in thyroid neoplasms [96]. Some of the antibodies that have been examined include galectin-3, Hector Battifora mesothelial cell antibody (HBME-1), cytokeratin-19, *RET*, TTF-1, hTERT, telomerase, p27 and p53 to name a few (Table 2). Two markers that have been extensively studied are galectin-3 and HMBE-1. When each of these antibodies was individually examined, variable results were obtained. Some studies found that these markers could differentiate between benign and malignant thyroid lesions and that the protein expression varied as a function of the tumor stage of papillary thyroid cancer [97,98,99,100,101]. Although these markers supported the diagnosis for the classical type of papillary thyroid cancer, they did not help for its histological variants. On the other hand, when these markers were combined (e.g., galectin-3 + HBME-1; HBME-1 + CK-19; galectin-3 + HBME-1 + CK-19), several investigators reported improved sensitivity and specificity for the detection of cancer seen on tissue or FNA samples [101,102,103,104]. Consequently, these results suggest that panels or patterns of these and other markers may represent the future of analyzing protein expression to improve the diagnostic certainty of the indeterminate thyroid nodular lesion. 

**Table 2:** Significant IHC Markers in Differentiated Thyroid Cancer.

Marker	Expression in thyroid cancer	Marker	Expression in thyroid cancer
Galectin-3	Upregulated	HER4	Upregulated
CK19	Upregulated	TG	Downregulated
VEGF	Downregulated	MIB-1	Upregulated
Aurora-A	Upregulated	Caveolin	Upregulated
p16	Upregulated	Aurora-C	Upregulated
AR	Upregulated	S100	Upregulated
HBME-1	Upregulated	MRAS	Downregulated
Bcl-2	Downregulated	c-kit	Downregulated
Cyclin-D1	Upregulated	HER3	Upregulated
CAV-1	Upregulated	RET	Upregulated
Cyclin-E	Upregulated	AMF-R	Upregulated
E-CAD	Downregulated	MLH1	Downregulated
Clusterin	Upregulated	AAT	Upregulated
CR	Upregulated	TTF-1	Upregulated
IGFBP5	Upregulated	PGI	Upregulated
P21	Upregulated	HSP-27	Downregulated
IGFBP2	Upregulated	Syntrophin	Upregulated
CTNNB1	Upregulated		

**Bold = most discriminatory markers**; IHC = immunohistochemical. With kind permission from Springer Science+Business Media: *Annals of Surgical Oncology*, Molecular Phenotyping of Thyroid Tumors Identifies a Marker Panel for Differentiated Thyroid Cancer Diagnosis, Vol. 15, 2008, 2811–2826, Wiseman SM *et al*., Table 5, © 2008 The Society of Surgical Oncology, Inc.

### 6.2. High-Throughput Proteomics

The study of biomarkers has now expanded to include the analysis of the proteome, which investigates protein expression and modification, along with change in protein activity and protein localization [3]. The information gained from the proteome reflects not only the expressed genomic profile of the cell, but also takes into account post-translational changes that are not detected at the mRNA level, as well as protein expression at different time points along a cell’s spectrum of neoplastic transformation [3,95,105]. The advantage of proteomics is the ability to detect biomarkers from the patient’s serum or plasma. The idea that a tumor is capable of “leaking” proteins into the circulation ultimately allows potential serum biomarkers to be identified [106]. 

The study of proteomics uses high-throughput technologies, which combines multidimensional separation systems based on mass spectrometry analysis and ProteinChip technology (Ciphergen Biosystems, USA) to improve sensitivity and specificity of a complex mixture of proteins and peptides from either tissue or serum [107,108]. Currently there are two proteomic methods for identifying biomarkers. The first method uses mass spectrometry (MS) to identify protein patterns by determining the mass:charge ratio and amino-acid sequence of proteins within a given specimen. When MS is combined with an ionization technique, such as matrix-assisted laser desorption ionization-time of flight (MALDI-TOF), surface-enhanced laser desorption/ionization-time of flight (SELDI-TOF) or electrospray ionization, it improves the sensitivity and rapid detection of cancer-specific biomarkers and proteomic-profile patterns [3,108,109]. The second method uses the technique of proteolytic digestion by exposing the protein samples to high efficiency liquid chromatography along with tandem MS analysis. This technique improves both protein identification and abundance, which translates into detecting and identifying larger amounts of peptides from complex protein mixtures [109,110]. Once MS has established a protein profile pattern, this data is read by artificial intelligence-based bioinformatics systems that serve as diagnosticians and help discriminate between normal, benign, pre-malignant, or malignant disease from a patient’s serum or tissue [108]. 

The first study that established a proteomic profile of benign and malignant human thyroid tissue was reported in 2002 [111]. In that study, cathepsin B was found to be up-regulated in the neoplastic tissues of follicular adenoma, follicular carcinoma, and papillary carcinoma when compared to non-neoplastic thyroid tissue. Unfortunately, cathepsin B could not distinguish between follicular adenoma and follicular carcinoma. The study also detected the up-regulation of two proteins within papillary thyroid cancer: ATP synthase D chain (ATPQ) and prohibitin (PHB) [111]. The clinical and therapeutic usefulness of these findings are still unknown. 

Two subsequent studies using two-dimensional difference gel electrophoresis (2D-DIGE) and MALDI-TOF mass spectrometry attempted to delineate a set of thyroid biomarkers through protein analysis. Brown *et al.* [112] compared the protein expression profiles of tissue from papillary thyroid cancer (PTC) specimens with matched normal thyroid tissue from the same patients and normal thyroid tissue from benign follicular adenomas. Using statistical validation software they discovered three novel biomarkers for PTC: (1) S100A6 (an isoform of S100 protein); (2) peroxiredoxin 2, and (3) heat shock protein 70 (HSP70), all three of which showed more than a two-fold difference in protein expression. In addition, they also confirmed the overexpression of known PTC biomarkers as identified by mRNA expression, which included galectin-3, cytokeratin-19 and cathepsin-B. Interestingly, they found two distinct forms of cathepsin-B in PTC, one form that was under expressed and another form (more acidic) that was over-expressed. The authors noted that cathepsin-B is involved in thyroglobulin expression and it is well known that serum thyroglobulin is a marker for disease recurrence. The differences seen in the different forms of cathepsin-B protein expression reflect the need for further study of these post-translational changes to validate these proteins as thyroid cancer biomarkers [112]. The second study, conducted by Netea-Maier *et al.* [113], examined protein abundance differences between thyroid follicular adenomas and follicular carcinomas. They discovered a statistically significant difference in protein abundance among 43 proteins between follicular thyroid carcinoma and follicular adenoma. Based upon previous reports they decided to examine three proteins (HSP gp96, PDI A3 and calreticulin) in greater detail and subsequently performed immunohistochemical analysis on a different set of paraffin-embedded tissue blocks for validation purposes [113]. These three proteins (HSP gp96, PDI A3 and calreticulin) are involved in protein folding and consequently a subset (HSP gp96 and calreticulin) may play a role in anti-cancer immunogenicity [114,115]. Immunohistochemical analysis revealed that calreticulin was the best single marker for distinguishing between follicular adenoma and follicular thyroid carcinoma, and all three markers together showed a high sensitivity at predicting widely invasive follicular thyroid carcinoma [113]. Because the discovery proteomics and the immunohistochemical validation studies proved to be accurate for distinguishing between follicular adenoma and follicular thyroid carcinoma for these three markers, prospective studies are now needed to prove the clinical utility of this approach.

To expand upon the study of proteomics, Giusti *et al.* [116,117] was the first group to analyze the fluid obtained from fine needle aspirates (FNA) of thyroid cancer. They used a combination of 2D-GE and MALDI-TOF-MS analysis to acquire a proteomic profile of a variety of classes of proteins from an FNA thyroid biopsy. In a follow-up study, they then examined the proteomic profiles of FNA samples from papillary thyroid tumors (comparing the classical and tall cell variants) as well as control FNA specimens from the opposite (unaffected) thyroid lobe. They discovered 17 protein spots that could reliably distinguish between papillary thyroid cancer and normal thyroid tissue [117]. Some of these proteins had been previously linked to thyroid cancer by other methods [111,112,118,119]. The proteomic analysis also showed a difference in protein expressed between the classical variant of PTC and the tall cell variant of PTC. In particular, ferritin heavy chain [FHC], peroxiredoxin 1 [PRX1] and 6-phosphogluconate dehydrogenase [6-PDGH] were exclusively upregulated in the tall cell variant of papillary thyroid cancer [117].

The most recent proteomic studies on thyroid cancer utilized SELDI-TOF mass spectrometry to distinguish between PTC and benign thyroid disease based upon serum protein profiles [107,120]. The major advantage of the SELDI-TOF technique is that it uses minute amounts of serum, coupled to bioinformatics systems that allow for high-throughput technology. According to Wulfkuhle [108], proteomic patterns or fingerprints, instead of protein identities, can generate discernable biomarker patterns. According to Wang [107], different protein expression factors can distinguish among four distinct categories: (1) PTC *vs.* normal thyroid tissue; (2) PTC *vs.* benign thyroid nodules; (3) various pathological stages of PTC; and (4) different pathological types of thyroid cancer. Thus, several biomarker patterns could be identified as a distinguishing feature within each category. Similarly, when three different groups were examined: (1) benign nodular disease *vs*. PTC; (2) benign nodular disease *vs.* controls; and (3) PTC *vs.* controls, a set of protein biomarkers distinguished benign nodular disease from PTC, based on different proteomic expression patterns, with a high degree of sensitivity and specificity (85.7%, 100%, respectively) [120]. Fan *et al.* [121] utilized several proteomic techniques to identify and validate their findings. The proteomic profiles of serum samples from patients diagnosed with PTC and non-cancer controls were analyzed. SELDI-TOF-MS technology was used to screen for potential candidate protein patterns and high pressure liquid chromatography, tandem MS and ProteinChip immunoassays were used to purify, identify and confirm the protein biomarkers, respectively. They identified a set of three protein biomarkers (haptoglobin alpha-1 chain, apolipoprotein C-I and apolipoprotein C-III) capable of differentiating between PTC and non-cancer controls. Another study used tissue from three patients diagnosed with PTC [122]. Data analysis from three different protein chips identified approximately 63 protein biomarkers that could potentially distinguish between PTC and normal thyroid tissue from the same patients. The drawback of this study was that they failed to determine the identities of these 63 protein biomarkers (via SELDI-TOF-MS) and only three samples of PTC were examined. However, expanding this technique to a larger group of samples with different thyroid pathologies may help to identify a panel of protein biomarkers to help distinguish benign from malignant thyroid neoplasms.

More recent proteomic research in thyroid cancer has focused on discovering biomarkers with the potential to identify therapeutic targets. Neutrophil gelatinase-associated lipocalin (NGAL) is a protein involved in the inflammatory and immune response system and is expressed in the FRO thyroid cancer cell line (a human anaplastic thyroid cancer cell line). Through 2D-GE and MALDI-TOF analysis, NGAL was identified as a mediator of NF-κB, a protein responsible for oncogenic activity in poorly differentiated thyroid cancer cell lines [123]. In addition, NGAL may serve as a potential therapeutic target due to its regulatory activity of iron. NGAL is responsible for transporting iron from the extracellular space into the cell. Increased intracellular iron levels have been shown to contribute to excess DNA synthesis via multiple transcription factors leading to tumor development [124,125,126]. This information may help identify therapeutic targets against NGAL and its role in iron uptake because iron chelators are already in clinical use today.

In 2009, a glycoproteomic profile of biomarkers was obtained by using liquid chromatography-tandem mass spectrometry to identify cell surface and secreted glycoproteins [127]. Both well-differentiated (FTC-133, XTC-1, TPC-1) and undifferentiated (ARO, DRO-1) thyroid cancer cell lines were studied, and an average of 150 glycoproteins were identified for each cell line examined. Most of the glycoproteins were either cell surface proteins or secretory proteins. Interestingly, a subset of five cell membrane glycoproteins (vasorin, NCAM-1, trophoblast glycoprotein, discoidin and integrin alpha-5 chain) were only found in the well-differentiated thyroid cancer cell lines as compared to the undifferentiated anaplastic cell lines [127]. This information holds potential for developing therapeutic strategies at the level of cell surface, integral membrane, or secretory glycoproteins, but validation studies are still needed. 

A 2009 study reported the presence of *RET* oncogene mutations in human medullary thyroid cancer cell lines (TT and MZ-CRC-1) based on proteomic analysis and antibody-based validation techniques [128]. The study compared expression profiles of downstream signaling elements (e.g., *RAS*-mitogen-activated protein kinases [MAPKs], phosphatidylinositol 3-kinase [PI3K], c-jun N-terminal kinase [JNK]) from *RET*-MEN2A and *RET*-MEN2B expressing cells. There were 41 phosphotyrosine proteins detected downstream following *RET* activation from two different medullary thyroid cancer cell lines and the EGFR pathway was over-expressed. It is hoped that this information might provide new targets for anti-cancer therapies. The analysis of proteomics coupled with the analysis of gene array expression and epigenetic changes is unraveling the oncogenic pathways for the development of targeted therapeutics, as seen in breast, prostate and colorectal cancer [129].

## 7. Conclusions

The study and identification of biomarkers in thyroid cancer has a long history. From the beginning, the goal was to find markers that can identify benign and malignant thyroid tumors and to predict the behavior of these thyroid cancers. The evolution of thyroid biomarker research reflects the standard thinking of the time and has changed in parallel with the advancement of technology. Although thyroid cancer is one of the least deadly forms of cancer, research in the field has remained on the cutting edge of science and technology, but better diagnostic tests and predictors of tumor aggressiveness are necessary. “Finding a cure” for thyroid cancer should remain a strong impetus for continued research to identify new biomarkers and therapeutics for this disease. 

This review highlights the fact that even though a significant amount of progress has been made in cancer research, we are only at the beginning of fully understanding how normal cells progress to tumors. This is why many thyroid cancer biomarkers have been identified, but few have made it into routine clinical practice. Calcitonin and thyroglobulin are markers for disease recurrence after surgical resection, and the *RET* point mutations are used to predict susceptibility and prognosis of a patient with familial medullary thyroid cancer. None of the other biomarkers reviewed in this study are currently in routine clinical use, but are being used as building blocks for the future to improve treatment.

Biomarker research continues to advance. Serum-based markers are moving from proteins secreted by tumors to detecting minute amounts of genetic material being shed by the tumor into the circulation. DNA mutations are still being studied and discovered, and have progressed from single mutation discoveries to the synthesis of “mutation pathways” and panels of DNA mutations as a single “signature”. Epigenetic regulation has become a part of biomarker research, and will increase in use as a biomarker as our understanding of these processes improves. High-throughput technologies have advanced our ability to study the human genome, but much work in the field still needs to be done in the validation and quality control of the outcome data. These technologies have also greatly expanded our ability to study the proteome, going from the study of single proteins to identifying proteomic signatures of thousands of expressed proteins. We have also begun to see a crossover of the different disciplines. Proteomics is being applied to serum-based studies, genomics is helping to identify new DNA mutations, and epigenetic regulation has changed our understanding of how DNA regulation takes place. 

Thyroid biomarker investigations were once the realm of a single scientist working on elucidating the mechanisms and utility of a single biomarker. We are moving into an era of biomarker research that requires a multi-disciplinary team of biologists, geneticists, statisticians, oncologists, surgeons, and even engineers. This team has the task of understanding and interpreting data from new technologies and incorporating these findings into our understanding of tumor formation. It may be that a combination of serum based biomarkers, epigenetics, genomics, and proteomics will be required to produce reliable and clinically helpful biomarkers. It could also be that ideas and mechanisms that have not yet been discovered will be the key to thyroid biomarkers. New areas of study like metabolomics, the study of the end products of cellular processes, and the kinome, the full complement of human protein kinases, have not been studied in thyroid cancer. Thyroid biomarker discovery remains an exciting field of thyroid cancer research, and will remain on the cutting edge of technology.
